# Polygenic risk for schizophrenia predicting test-measured and self-reported cognitive performance in individuals without psychosis

**DOI:** 10.1186/s12888-026-07775-x

**Published:** 2026-01-12

**Authors:** Elena Rosenqvist, Leo-Pekka Lyytikäinen, Elina Sormunen, Mika Kähönen, Olli Raitakari, Jarmo Hietala, Katja Pahkala, Terho Lehtimäki, Liisa Keltikangas-Järvinen, Suvi Rovio, Aino Saarinen

**Affiliations:** 1 Department of Psychology, Faculty of Medicine, University of Helsinki, P.O. Box 21, Haartmaninkatu 3, Helsinki, 00014 Finland; 2 Faculty of Medicine and Health Technology, Tampere University, Tampere, Finland; 3 Department of Clinical Chemistry, Fimlab Laboratories, and Finnish Cardiovascular Research Center, Tampere, Finland; 4 Department of Cardiology, Heart Center, Tampere University Hospital, Tampere, Finland; 5 Department of Psychiatry, University of Turku and Turku University Hospital, Turku, Finland; 6 Department of Clinical Physiology, Tampere University Hospital and Faculty of Medicine and Health Technology, Tampere University, Tampere, Finland; 7 Research Centre of Applied and Preventive Cardiovascular Medicine, University of Turku, Turku, Finland; 8 Centre for Population Health Research, University of Turku and Turku University Hospital, Turku, Finland; 9 Department of Clinical Physiology and Nuclear Medicine, Turku University Hospital, Turku, Finland; 10 Department of Medicine, University of Turku and Division of Medicine, Turku University Hospital, Turku, Finland; 11 Department of Public Health, University of Turku and Turku University Hospital, Turku, Finland

**Keywords:** Cognitive function, Cognitive performance, Cognitive ability, Executive function, Psychosis

## Abstract

**Introduction:**

Schizophrenia is characterized by weaker test-measured cognitive performance, which is partially explained by disease-related secondary factors (after the onset of the disorder) such as side effects of antipsychotics, stigma, or sedentary behavior. We examined whether polygenic risk for schizophrenia (PRSSCZ) is associated with (a) test-measured or (b) self-reported cognitive performance in individuals who have not converted into non-affective psychosis during follow-up to extending to middle age.

**Methods:**

The participants came from the population-based Young Finns Study, born between 1962 and 1977 (*n* = 2217). Participants with diagnosed non-affective psychoses were excluded from the sample. Diagnoses collected from the Care Register for Health Care. PRS_SCZ_ was calculated on the basis of the most recent genome-wide association study on schizophrenia. Cognitive performance was measured with (1) subtests of the Cambridge Neuropsychological Test Automated Battery, measuring visuospatial learning, reaction time, sustained attention, and executive function and (2) self-reported executive functions including distractibility, task orientation, and rigidity.

**Results:**

In individuals who have not developed non-affective psychoses during follow-up to middle age, high PRS_SCZ_ was associated with lower scores in all test-measured cognitive domains. These associations sustained after controlling for health behaviors and socioeconomic factors. PRS_SCZ_ was not associated with self-reported distractibility or task orientation but was related to an increasing trajectory of rigidity when approaching middle age.

**Conclusion:**

We observed lower cognitive functioning in domains similar to those reported in studies of patients with schizophrenia. Thus, some difficulties in cognitive performance may not be fully attributable to the disorder itself but may partly reflect normative developmental trajectories in individuals with high polygenic liabilities.

**Clinical trial number:**

Not applicable.

**Supplementary Information:**

The online version contains supplementary material available at 10.1186/s12888-026-07775-x.

## Introduction

Schizophrenia is associated with lower performance across cognitive domains [1, 2]. To some degree, this is explained by disease-related secondary factors (after an onset of the disorder), such as side effects of antipsychotic medications [3], internalized stigma [4], long duration of untreated psychosis [5], negative symptoms (explaining ca. 10–15% of cognitive dysfunction) [6], or physical inactivity [7]. Therefore, research is needed whether lower cognitive performance is evident also among those who are at risk for psychosis but have not developed the disorder.

The strongest single risk factor for schizophrenia is familial risk, with approximately 9–10% of first-degree relatives of schizophrenia patients developing the disease during their lifetime [8]. Indeed, schizophrenia has a strong genetic background: the heritability of schizophrenia is estimated to be around 80% [9, 10]. Recently, genome-wide association studies (GWAS) on schizophrenia have provided possibilities to calculate polygenic risk scores for schizophrenia (PRS_SCZ_), including the single nucleotide polymorphisms (SNPs) associated with schizophrenia [11]. Polygenic risk scores for schizophrenia have been estimated to explain 8–33% of the variation in liability to schizophrenia [12–14]. Thus, a recent method to identify those at risk for psychosis is to calculate polygenic risk scores for schizophrenia.

To date, a few studies have examined cognitive performance among those who have high PRS_SCZ_ but who have not developed a non-affective psychosis. The results have been inconclusive. More specifically, some studies have reported correlations between high PRS_SCZ_ and lower general cognitive ability [15–17], weaker episodic memory, semantic memory, and visuospatial ability [18], weaker semantic fluency and verbal memory [19], and weaker social cognition [20] in healthy individuals. Other studies, in turn, have found no associations between PRS_SCZ_ and cognitive abilty in healthy individuals [18, 19, 21].

Essentially, test-based cognitive performance may not provide a comprehensive measure of individual’s cognitive functioning in individuals at risk for psychosis. Some studies have reported rather low correlations between cognitive test performance and cognitive functioning observed in every-day life [22]. Low correlations may be explained by, for example, under-achieving in a cognitive test situation due to temporary distractors such as distress or sleep disturbances [23]. Also, various compensation strategies can be adopted to maintain good cognitive performance in a test situation despite lower cognitive functioning [24]. As a result, many studies have expressed concerns about the ecological validity of cognitive tests [24, 25]. Thus, in parallel with cognitive tests, it is necessary to investigate also self-reported every-day cognitive functioning. In the context of psychotic disorders, self-reports are needed especially on executive functions because executive functioning has a strong effect on every-day functioning in individuals with prodromal psychosis [26] and because executive functioning has a central role in the utilization of other cognitive abilities [27]. As far as we know, however, no study has investigated the association of PRS_SCZ_ with self-reported cognitive functions in individuals who have not developed a non-affective psychosis.

This study investigated the associations of polygenic risk for schizophrenia (PRS_SCZ_) with test-measured and self-reported domains of cognitive performance in adults who have not developed a non-affective psychosis during follow-up to middle age. We used the population-based and prospective dataset of the Young Finns Study (YFS). Test-based cognitive performance was assessed using the Cambridge Automated Neuropsychological Test Battery (CANTAB), measuring visuospatial learning, reaction time, sustained attention, and executive function. Self-reported cognitive functions were examined in terms of rigidity, task orientation, and distractibility. We also considered possible confounders such as health behaviors and socioeconomic factors.

## Methods

###  Participants

The participants are part of the Young Finns Study (YFS) which is an ongoing prospective follow-up study that started in 1980. The original study population was selected from the population register of the Social Insurance Institution and included 3569 participants from six age cohorts (born in 1962, 1965, 1968, 1971, 1974 and 1977). All the participants were non-institutionalised and, thus, most severe cognitive disabilities were excluded from the sample. The baseline participants have been followed in 1983, 1986, 1989, 1992, 1997, 2001, 2007, 2011/2012, and 2018–2020. The population of the YFS is described in further detail elsewhere [28]. The YFS has been approved by the Ethics Committees of five Finnish Universities (Universities of Helsinki, Turku, Tampere, Kuopio, and Oulu). Informed consent was obtained prior to participation from all the participants or, if a participant was under 18 years old, from his/her parents. The study has been conducted in accordance with Declaration of Helsinki.

In this study, we included participants (*n* = 2217) with data available on polygenic risk for schizophrenia, psychiatric diagnoses (no missing values), test-measured cognitive performance in 2011, self-reported cognitive functions in 1997 or 2001, and control variables (childhood SES in 1980, and adulthood SES and health behaviors in 2011).

### Measures

#### Polygenic risk score for schizophrenia

Polygenic risk score for schizophrenia was calculated using PRS-CS method [29], which infers posterior SNP effect sizes under continuous shrinkage (CS) priors using GWAS summary statistics and an external LD reference panel. The latest available schizophrenia GWAS results [30] were used as SNP summary statistics and HapMap 3 EUR as an external LD reference [31]. Variants with minor allele frequency (MAF) ≥ 0.01 and imputation information ≥ 0.3 were included in the PRS calculation.

Genotyping was done for 2556 samples using custom build Illumina Human 670k BeadChip at Welcome Trust Sanger Institute. Sample call rate < 0.95, excess heterozygosity, sex mismatch, cryptic relatedness (ˆπ > 0.2), SNP call rate < 0.95, MAF < 0.01, and HWE p-value < 1e − 6 were used as quality control filters. After quality control, there were 2443 samples and 546,677 genotyped SNPs available for further analysis. Genotype Imputation to TOPMed r3 reference was performed using TOPMed Imputation Server with Minimac 4.

#### Test-measured cognitive performance

Cognitive performance was measured in 2011 using the CANTAB (Cambridge Neuropsychological Test Automated Battery) tests [32]. CANTAB is a computerized, non-linguistic, and culturally neutral test battery consisting of 24 subtests of wide range of cognitive functions. In this study, four subtests of the CANTAB were used: Paired Associates Learning test (PAL) is used to assess visuospatial associative learning and visual episodic memory [33]. The Reaction Time test (RTI) assesses reaction time and response accuracy [34]. The Rapid Visual Information Processing test (RVP) measures sustained (visual) attention, visual processing, and visual recognition [35]. The SWM test is used to assess executive function, including abilities to retain information from spatial working memory and to use self-organized search strategies in problem-solving [36]. These four subtests have previously been found to have adequate to good concurrent validity with pen-and-paper cognitive tests measuring similar cognitive functions [35] and adequate to high test-retest reliability [34].

The CANTAB variables were constructed by calculating sum variables for each outcome variable of each cognitive test (e.g., reaction time, number of errors and movement time for RTI), and standardizing them into a scale with a mean of 0 and SD of 1. Test-specific scores were then calculated by summing the standardized variables of each subtest and dividing the sum by the number of variables within each subtest. A detailed description of the cognitive performance testing and the calculation of the CANTAB variables are described elsewhere [37].

#### Self-reported cognitive functions

Self-reported cognitive functions were assessed in 1997 and 2001 using self-report scales of rigidity, task orientation, and distractibility. The measures of self-reported cognitive functions were obtained from the DOTS-R (the Revised Dimensions of Temperament Survey) questionnaire. The scales included five statements on rigidity (e.g., “Changes in plans make me restless”) and distractibility (e.g., “When I am doing something, nothing else can distract me”) and eight statements on task orientation (e.g., “I continue completing the task until I get it done”). The statements were responded with a 5-point Likert scale (1 = totally disagree, 5 = totally agree). We calculated mean scores for the self-reported cognitive functions. First, mean variables for each cognitive function were calculated for each measurement point (1997 and 2001) for those participants who had responded to > 50% of the statements of the scale. Next, we calculated a mean score for each self-reported cognitive function between the measurement years of 1997 and 2001 (for those participants who had data available in at least one measurement year).

The same scales are previously found to predict paranoid ideation in this dataset [38], supporting their predictive validity in the context of psychotic disorders. Further, in offspring of schizophrenia patients, high rigidity scores are associated with increased risk of internalising and externalising disorders [39]. Further, the internal reliabilities of the scales were adequate in our sample (Cronbach’s alpha = 0.79 for task orientation, 0.79 for distractibility, and 0.70 for distractibility).

#### Psychiatric diagnoses

In this study, we excluded participants who had developed a non-affective psychosis during follow-up to middle age. For that purpose, psychiatric diagnoses until year 2017 were collected from the Care Register for Health Care, covering all psychiatric disorders that have required hospital care. In 2017, the participants were 40–55 years of age which is clearly over the typical onset age of schizophrenia [40]. The diagnoses were given in accordance with the existing diagnostic classification (ICD-8, ICD-9, or ICD-10). The ICD-diagnoses were then converted into DSM-diagnoses; this convertion is described with further details elsewhere [41]. The diagnoses were then classified into non-affective psychotic disorders, substance-related disorders, affective disorders (mood and anxiety disorders), and personality disorders. As we excluded participants with diagnosed non-affective psychoses, the register was well-suitable for that purpose since the register is found to cover 93% of schizophrenia-spectrum psychoses and 97% of psychotic disorders [42].

#### Control variables

The analyses were adjusted by sex, age, health behaviors, and/or childhood and adulthood SES, all of which are known to be associated with cognitive performance [43–46]. Health behaviors were measured by leisure-time physical activity, alcohol consumption, and daily smoking status. For leisure-time physical activity, a continuous sum variable was constructed, assessing the frequency, number of hours and duration of leisure-time physical activity. The index is described in further detail elsewhere [47]. Alcohol consumption was measured as a continuous variable indicating the number of doses of alcohol the person reported consuming per week. One dose was defined as a 0.3 l cans or bottles of beer, 12 cl of wine, and 4 cl shots of liquor or strong alcohol [48]. Daily smoking status was assessed as a dichotomous variable that indicated whether the respondent smoked daily (yes / no). Childhood SES was measured in 1980 by parental education (comprehensive school / college level / academic level) and a continuous variable of parental income ranging from 15 000 to 100 000 Finnish marks (the former currency of Finland). We controlled for childhood SES in 1980 because, first, we had the broadest data available at baseline, and second, previous studies on this same dataset have found significant effects of the childhood SES on cognitive performance [49]. Adulthood SES was measured by education (comprehensive school / college level / academic level) and a continuous income variable ranging from 5 000 euros to 60 000 euros.

### Statistical analyses

The analyses were performed using STATA/MP 18. First, participants with diagnosed non-affective psychotic disorders were excluded from the data in order to investigate those who have not converted to psychosis. Second, we calculated pairwise correlations between the study variables.

Then, we investigated the associations of PRS_SCZ_ with test-measured and self-reported domains of cognitive performance using regression analyses. The analyses consisted of two parts. First, we predicted each CANTAB domain (PAL, RTI, RWI and SWM) separately by PRS_SCZ_. Second, we predicted the three self-reported domains of cognitive functions (rigidity, task orientation, and distractibility) separately by the PRS_SCZ_. For each analysis, we had three different models by adding control variables in a stepwise manner: Model 1 was adjusted for sex and age, Model 2 also for childhood and adulthood SES (parents’ education and annual income, and participants’ education and annual income as four separate variables), and Model 3 also for adulthood health behaviors (alcohol consumption, smoking, physical activity as three separate variables). As there were no significant sex-interactions of PRS_SCZ_ when predicting test-measured or self-reported domains of cognitive performance or self-reported cognitive functions, the analyses were performed for both sexes simultaneously.

Finally, we used false discovery rate correction (FDR) for multiple testing.

## Results

Descriptive statistics are presented in Table 1. Approximately 55% of the participants were female, and 75.5% of the participants had an academic-level education. Pairwise correlations between the study variables are presented in Supplementary Table 1.

**Table 1:** Descriptive statistics of the study variables

Variable	Mean (SD)	Frequency (%)	Range
PRS for schizophrenia	-0.01 (1.00)		-4.72; 2.94
Cognitive test performance			
Visuospatial learning	0.01 (0.99)		-3.40; 1.91
Reaction time	0.01 (1.00)		-3.17; 2.20
Sustained attention	0.03 (0.99)		-2.20; 3.01
Executive function	0.01 (0.99)		-3.42; 2.12
Self-reported cognitive functions			
Rigidity	2.08 (0.58)		1.00; 4.20
Task orientation	2.76 (0.56)		1.00; 4.63
Distractibility	3.01 (0.67)		1.00; 4.90
Sex (Female)		1226 (55.3)	
Age (2001)	41.60 (5.05)		34; 49
Parental education			
Comprehensive school		713 (32.7)	
College level		906 (41.5)	
Academic level		565 (25.9)	
Parental income	4.89 (1.94)		1; 8
Adulthood education			
Comprehensive school		36 (2.2)	
High school or occupational school		372 (22.3)	
Academic level		1257 (75.5)	
Adulthood income	7.41 (3.03)		1; 13
Physical activity (2011)	9.06 (1.88)		5; 15
Alcohol use (2011)	0.80 (1.10)		0; 10
Daily smoking status (2011)		241 (14.3)	

*n* = 2217. Participants in at least one of the analyses are included

### Main results

**Fig. 1:**
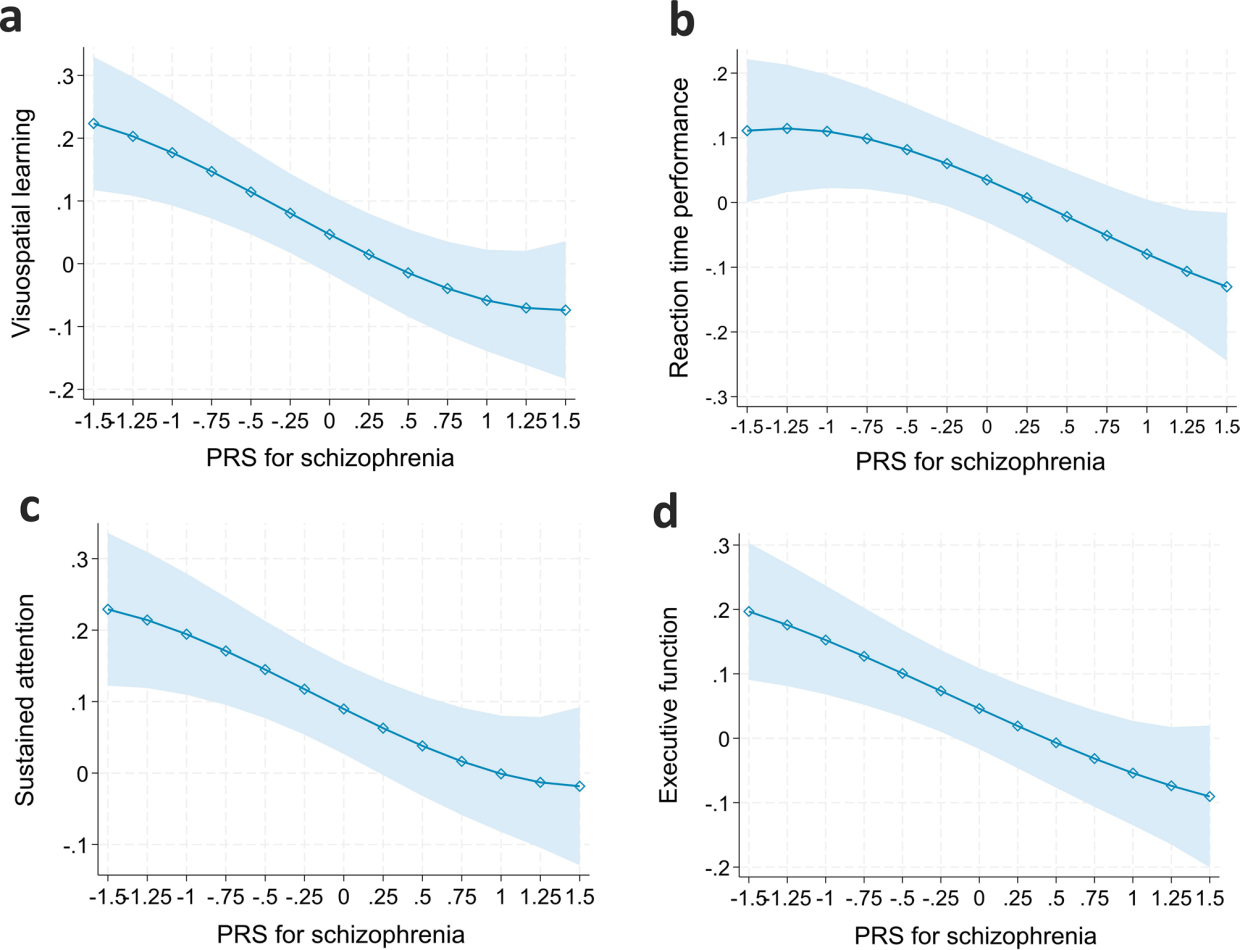
In individuals without non-affective psychotic disorders: model-predicted values of test-measured (**a**) visuospatial learning, (**b**) reaction time, (**c**) sustained attention, and (**d**) executive function at different levels of PRS_SCZ_ (Z-score, mean = 0, SD = 1). Note: Adjusted for age, sex, childhood and adulthood SES, and adulthood health behaviors

Table 2 shows the results from linear regression models predicting test-measured cognitive performance. When adjusting for age and sex (Model 1), high PRS_SCZ_ was associated with weaker visuospatial learning (B = -0.061, *p* = 0.0018), slower reaction time (B = -0.086, *p* = 0.015), weaker sustained attention (B = -0.086, *p* = 0.00067), and weaker executive function (B = -0.076, *p* = 0.00043). All the associations remained significant after adjusting also for socioeconomic factors (Models 2) and after adjusting also for adulthood health behaviors (Models 3). All the associations also sustained after FDR correction. The results are illustrated in Fig. 1. The R squared values ranged between 0.07 and 0.10, 0.04‒0.05, 0.02‒0.09, and 0.07‒0.09 for visuospatial learning, reaction time, sustained attention, and executive function, respectively. Thus, the highest R squared values were found for visuospatial learning and executive function.

**Fig. 2:**
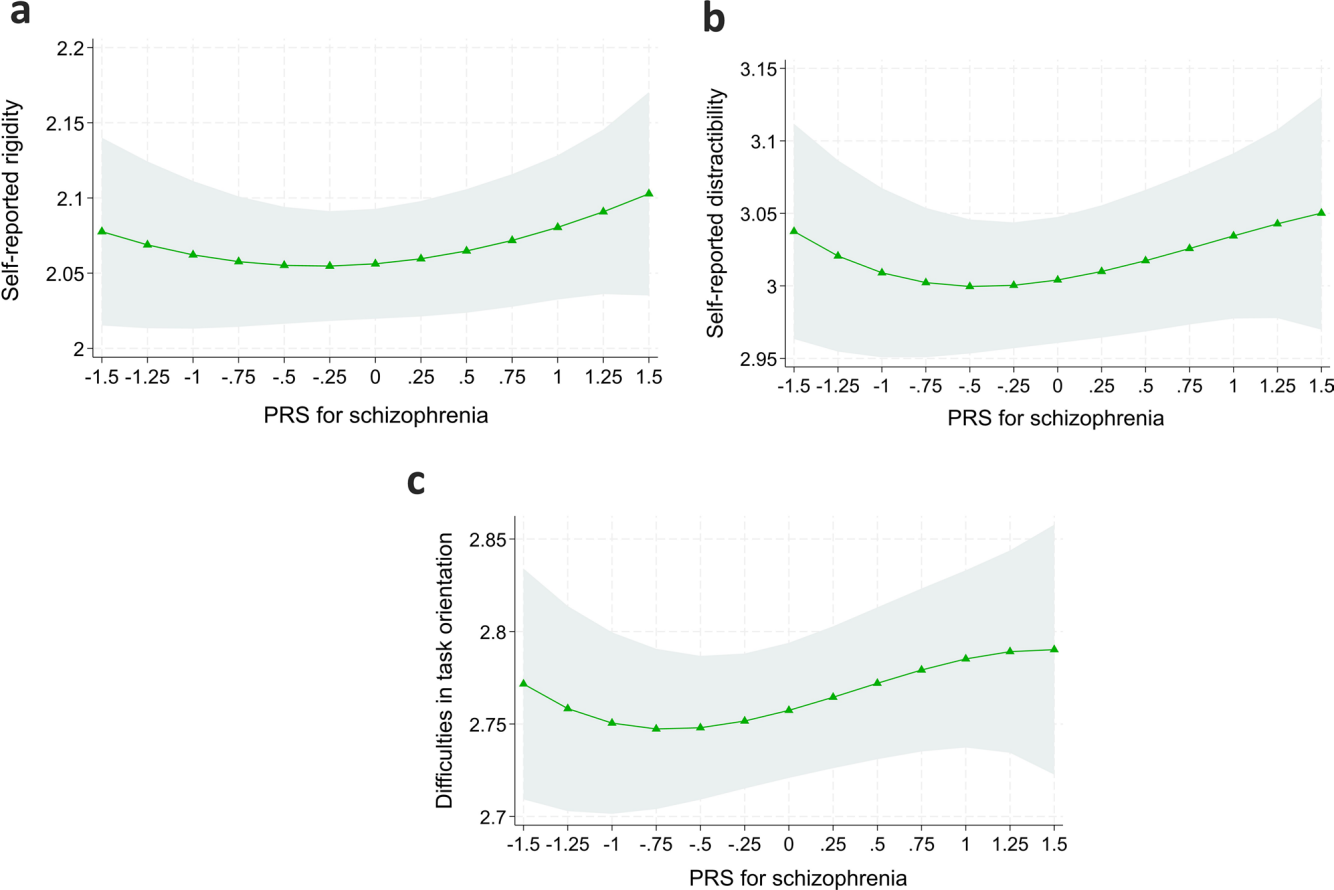
In individuals without non-affective psychotic disorders: model-predicted values of self-reported (**a**) rigidity, (**b**) distractibility, and (**c**) task orientation separately with high (highest 25% in the sample) or low (lowest 25% in the sample) PRS_SCZ_. Note: Adjusted for age, sex, childhood and adulthood SES, and adulthood health behaviors

The results of linear regression models predicting self-reported cognitive functions are presented in Table 3. PRS_SCZ_ was not associated with any domain of the self-reported cognitive functions, i.e., rigidity (*p* = 0.142–0.893, Models 1‒3), task orientation (*p* = 0.654–0.881, Models 1‒3), or distractibility (*p* = 0.607–0.831, Models 1‒3). All the associations also remained non-significant after FDR correction. The results are illustrated in Fig. 2.

**Table 2:** Main effect of PRS_scz_ on test-measured cognitive performance in individuals without non-affective psychotic disorders

		Visuospatial learning						Reaction time							Sustained attention							Executive function			
	B		SE	*p*	FDR-corr.		B		SE	*p*	FDR-corr.		B			SE	*p*	FDR-corr.		B			SE	*p*	FDR-corr.
Model 1	-0.076		0.024	0.0018	0.0063		-0.061		0.025	0.015	0.0315		-0.086			0.025	0.00067	0.0047		-0.086			0.024	0.00043	0.0047
Model 2	-0.075		0.026	0.0034	0.010		-0.064		0.027	0.017	0.0325		-0.068			0.026	0.0082	0.019		-0.083			0.026	0.0013	0.0055
Model 3	-0.089		0.026	0.0010	0.0053		-0.063		0.027	0.020	0.035		-0.073			0.026	0.0057	0.015		-0.092			0.026	0.00051	0.0047

Model 1 (*n* = 1543) is adjusted for age and sex

Model 2 (*n* = 1355) is adjusted for age, sex and childhood and adulthood SES. Model 3 (*n* = 1281) is adjusted for age, sex, childhood and adulthood SES, and adulthood health behaviors. SE = standard error FDR-corr. = false discovery rate corrected p value

**Table 3:** Main effect of PRS_scz_ with self-reported cognitive functions in individuals without non-affective psychotic disorders

		Rigidity					Difficulties in task orientation						Distractibility			
		B	SE	*p*	FDR-corr.		B	SE	*p*	FDR-corr.			B	SE	*p*	FDR-corr
Model 1		0.019	0.013	0.142	0.229		-0.003	0.012	0.835	0.893			-0.004	0.015	0.791	0.893
Model 2		0.009	0.015	0.570	0.850		-0.002	0.015	0.881	0.893			-0.004	0.018	0.831	0.893
Model 3		0.002	0.015	0.893	0.893		-0.007	0.015	0.654	0.858			-0.009	0.018	0.607	0.850

Models 1 (*n* = 2005) was adjusted for age and sex

Models 2 (*n* = 1382) were adjusted also for childhood and adulthood SES. Models 3 (*n* = 1309) were adjusted also for adulthood health behaviors.SE = standard error FDR-corr. = false discovery rate corrected p value

### Sensitivity analyses

As sensitivity analyses, we reran the analyses so that participants with any diagnosed psychiatric disorder (having required hospital care) were excluded from the sample. The remaining sample consisted of 1235‒1478 participants in Models 1‒3, respectively. All the results remained. Specifically, high PRS_SCZ_ was again associated with lower test-measured visuospatial learning (*p* = 0.002‒0.010 in Models 1–3), lower reaction time (*p* = 0.036‒0.038), lower sustained attention (*p* = 0.001‒0.007), and lower executive function (*p* = 0.001‒0.002). In addition, all the associations between PRS_SCZ_ and self-reported cognitive functions remained non-significant.

Next, we examined whether the associations of PRS_SCZ_ with cognitive performance could be modified by a diagnosis of non-affective psychosis, i.e., whether the associations could be different in participants with vs. without a non-affective psychosis. No significant interactions were found between PRS_SCZ_ and non-affective psychosis when predicting cognitive performance. However, this result should be interpreted with caution, as there were fewer than 100 cases of non-affective psychosis, resulting in limited statistical power.

**Fig. 3:**
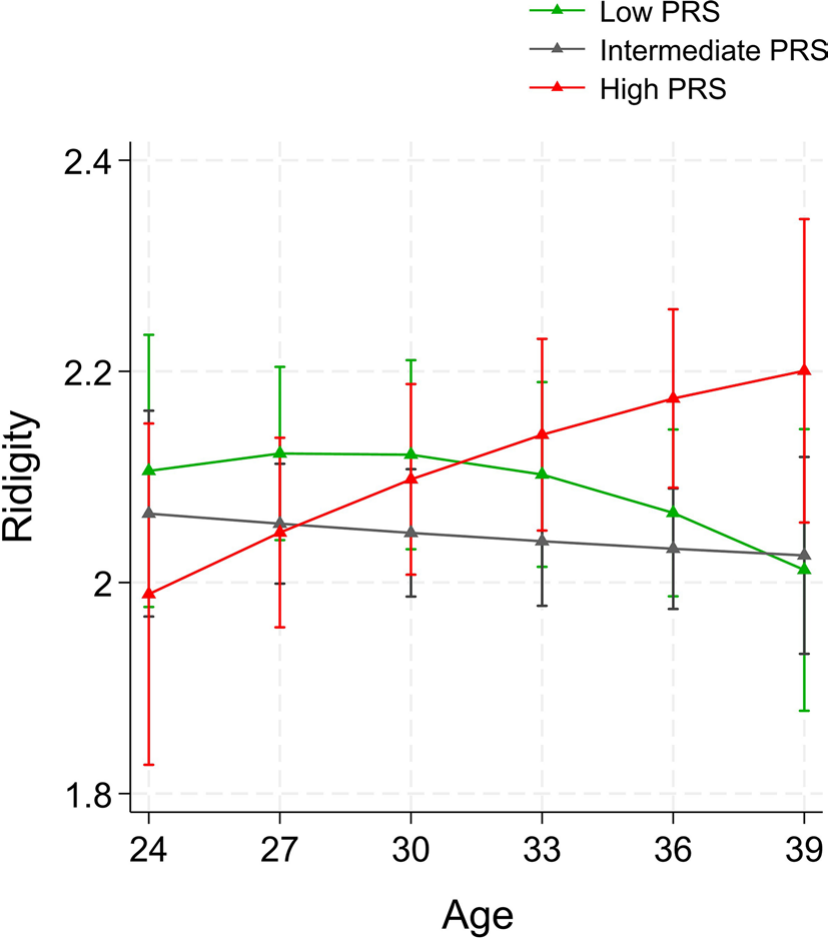
In individuals without non-affective psychotic disorders: model-predicted values of self-reported (**a**) rigidity, (**b**) distractibility, and (**c**) task orientation separately with high (highest 25% in the sample), intermediate, or low (lowest 25% in the sample) PRS_SCZ_. Note: Adjusted for age, sex, childhood and adulthood SES, and adulthood health behaviors

Finally, we examined age-interactions with PRS_SCZ_ when predicting test-measured and self-reported cognitive functions. There were not significant age-interactions with PRS_SCZ_ when predicting test-measured cognitive performance or self-reported task orientation or distractibility. However, we found that age modified the associations between PRS_SCZ_ and rigidity (*p* = 0.001‒0.013 in Models 1‒3). This interaction is plotted in Fig. 3. Thus, individuals with high PRS_SCZ_ seemed to have an increasing trajectory of rigidity over age, contrary to those with low or intermediate PRS_SCZ_.

## Discussion

We examined whether polygenic risk for schizophrenia (PRS_SCZ_) is associated with test-measured and self-reported cognitive performance in individuals who have *not* developed a non-affective psychosis during follow-up to middle age. In test-measured cognitive assessments, high PRS_SCZ_ was related to lower scores in all investigated domains, namely visuospatial learning, reaction time, sustained attention, and executive function. These associations remained significant after adjusting for health behaviors and socioeconomic factors. In the models, the highest R squared values were found for visuospatial learning and executive function with the values between 0.07 and 0.10. PRS_SCZ_ was not associated with self-reported task orientation or distractibility, but high PRS_SCZ_ was related to an increasing trajectory of self-reported rigidity over age. Overall, our study indicates that individuals with high PRS_SCZ_ have weaker test-measured performance in some cognitive domains, and they may experience an increasing trajectory of rigidity in their daily life as they approach middle age.

As mentioned before, a part of the schizophrenia-related cognitive alterations are known to be explained by secondary disorder-related factors such as side effects of antipsychotics [3] or strong stigmatization [4]. We found an association between high PRS_SCZ_ and weaker test-measured cognitive performance also among those who have not developed a psychosis until middle age. Overall, the results suggest that psychosis-related cognitive alterations may also reflect genetic liabilities that are unrelated to the onset of the disease process. Indeed, some studies have suggested that PRS_SCZ_ has some degree of genetic overlap with cognitive performance [50, 51]. Further, a previous meta-analysis and review concluded that PRS_SCZ_ may be even more strongly associated with cognition in general population than in schizophrenia patients [17]. However, while some studies have suggested that the association of PRS_SCZ_ with cognitive ability is male-specific [18, 19], we did not find any sex-interactions.

As far as we know, our study was the first one to investigate the association between PRS_SCZ_ and self-reported cognitive functions in healthy individuals. In our study, PRS_SCZ_ had no associations with self-reported task orientation or distractibility, but high PRS_SCZ_ was related to an increasing trajectory of self-reported rigidity when approaching middle age. Interestingly, schizophrenia patients are shown to have lower executive function in middle age than in younger ages [52]. This finding further supports an idea that individuals who have not developed non-affective psychosis may experience similar age-related trajectories of executive functioning. In our study, the rigidity scale assesses the disposition to become restless following changes in plans or schedules, as well as difficulties in adapting to variations in daily routines. Accordingly, rigidity is not only crucial in the light of cognitive functions but also plays a crucial role in interpersonal contexts and formation of social relationships [53].

A few methodological issues are necessary to be taken into consideration. First, CANTAB test battery has been designed to capture variance in non-clinical populations. Thus, in our general population sample, “lower performance” in the CANTAB tests should not be directly interpreted as cognitive “impairment”. Second, our CANTAB test battery did not include a test for verbal ability/memory. A decline in verbal memory is one of the most consistent findings of cognitive difficulties in schizophrenia patients [1, 54]. Third, the results on test-measured vs. self-reported cognitive performance were not fully comparable because they were measured in different follow-ups: cognitive test performance was assessed at the age of 34‒49 years, while self-reported cognitive functions were evaluated at the age of 20‒39 years. All measurements, however, were conducted in adulthood/midlife when cognitive abilities are found to be quite stable [55].

## Conclusion

In conclusion, the results provide evidence that high PRS_SCZ_ is associated with lower visuospatial learning, reaction time, sustained attention, and executive function in those who have *not* developed a non-affective psychosis. Further, individuals with high PRS_SCZ_ may experience increasing levels of rigidity in their daily life when approaching middle age. Thus, lower cognitive functions may not be fully explained by the onset of a psychotic disorder but may rather reflect a normative developmental trajectory of high polygenic liabilities to schizophrenia. Further, the results highlight the importance of using both test-based and self-report-based measures of cognitive performance among individuals at genetic risk for psychosis. In practice, it is essential to note that even individuals who have not developed a non-affective psychosis but who have a high genetic risk for schizophrenia, may benefit from support or cognitive training in order to promote their cognitive health.

## Supplementary Information

Below is the link to the electronic supplementary material.

Supplementary Material 1

## Data Availability

The Cardiovascular Risk in Young Finns (YFS) dataset comprises health-related participant data, and their use is therefore restricted under the regulations on professional secrecy (Act on the Openness of Government Activities, 612/1999) and on sensitive personal data (Personal Data Act, 523/1999, implementing the EU data protection directive 95/46/EC). Due to these legal restrictions, the data from this study cannot be stored in public repositories or otherwise made publicly available. However, data access may be permitted on a case by case basis upon request. Data sharing outside the group is done in collaboration with YFS group and requires a data-sharing agreement. Investigators can submit an expression of interest to the chairman of the publication committee (Prof. Mika Kähönen, Tampere University, Finland, http://www.mika.kahonen@tuni.fi.
