# Supplementary material for: Polygenic risk for schizophrenia predicting test-measured and self-reported cognitive performance in individuals without psychosis
Source: BMC Psychiatry. 2026 Jan 12;26:34. doi: 10.1186/s12888-026-07775-x (PMC12797622; doi:10.1186/s12888-026-07775-x)
Supplement: Supplementary file 1 — Supplementary Material 1 [file 12888_2026_7775_MOESM1_ESM.docx]

**Supplementary Table 1**. Pairwise correlations between the study variables. *Note:* An asterisk indicates statistical significance at *p* < 0.05.

|  | **1.** | **2.** | **3.** | **4.** | **5.** | **6.** | **7.** | **8.** | **9.** | **10.** | **11.** | **12.** | **13.** | **14.** | **15.** | **16.** | **17.** |
| --- | --- | --- | --- | --- | --- | --- | --- | --- | --- | --- | --- | --- | --- | --- | --- | --- | --- |
| **1. PRS_SCZ_** | - |  |  |  |  |  |  |  |  |  |  |  |  |  |  |  |  |
| **2. Sex** | .01 | - |  |  |  |  |  |  |  |  |  |  |  |  |  |  |  |
| **3. Age** | .00 | -.02 | - |  |  |  |  |  |  |  |  |  |  |  |  |  |  |
| **4. Visuospat. learning** | -.09* | .03 | -.26* | - |  |  |  |  |  |  |  |  |  |  |  |  |  |
| **5. Reaction time** | -.07* | -.12* | -.14* | .14* | - |  |  |  |  |  |  |  |  |  |  |  |  |
| **6. Sustained attention** | -.10* | -.07* | -.11* | .27* | .18* | - |  |  |  |  |  |  |  |  |  |  |  |
| **7. Executive function** | -.08* | -.14* | -.23* | .27* | .16* | .28* | - |  |  |  |  |  |  |  |  |  |  |
| **8. Rigidity** | .03 | .01 | .06* | -.06* | -.09* | -.06* | -.06* | - |  |  |  |  |  |  |  |  |  |
| **9. (Low) task orientation** | .00 | .14* | .03 | -.05 | -.05 | -.03 | -.04 | .11* | - |  |  |  |  |  |  |  |  |
| **10. Distractibility** | -.01 | .15* | .06* | -.06* | -.04 | -.04 | -.06* | .07* | .93* | - |  |  |  |  |  |  |  |
| **11. Parental education** | -.04* | -.02 | -.27* | .19* | .07* | .21* | .10* | -.09* | -.02 | -.01 | - |  |  |  |  |  |  |
| **12. Parental income** | -.04* | .00 | -.02 | .07* | .02 | .18* | .04 | -.04 | .01 | .01 | .48* | - |  |  |  |  |  |
| **13. Education** | -.03 | .16* | -.12* | .15* | .04 | .16* | .08* | -.08* | -.06* | -.05* | .22* | .18* | - |  |  |  |  |
| **14. Income** | .03 | -.31* | .04 | .08* | .11* | .20* | .10* | -.19* | -.17* | -.17* | .15* | .19* | .16* | - |  |  |  |
| **15. Physical activity** | -.01 | .05 | -.07* | .04 | .09* | .09* | .01 | -.08* | -.06* | -.01 | .08* | .06* | .11* | .08* | - |  |  |
| **16. Alcohol use** | .03 | -.30* | .11* | .00 | -.01 | .03 | .03 | .05 | .03 | .03 | .02 | .00 | -.12* | .10* | -.07* | - |  |
| **17. Daily smoking** | .04 | -.05 | -.02 | .00 | -.06* | -.08* | -.03 | -.01 | .06* | .05 | -.11* | -.09* | -.21* | -.08* | -.18* | .24* | - |
| n = 2217 | | | | | | | | | | | | | | | | | |
